# Magnitude of malnutrition and associated factors among HIV infected children attending HIV-care in three public hospitals in East and West Gojjam Zones, Amhara, Northwest, Ethiopia, 2017: a cross-sectional study

**DOI:** 10.1186/s13104-018-3882-8

**Published:** 2018-11-03

**Authors:** Yihenew Sewale, Getachew Hailu, Mizinew Sintayehu, Nurilign Abebe Moges, Animut Alebel

**Affiliations:** 1 Nursing Department, Medicine and Health Sciences College, Debre Berhan University, Debre Berhan, Ethiopia; 2 Nuring Department, Medicine and Health Sciences College, Debre Markos University, Debre Markos, Ethiopia; 3 Public Health Department, Medicine and Health Sciences College, Debre Markos University, Debre Markos, Ethiopia

**Keywords:** Malnutrition, Magnitude, Associated factors, Ethiopia

## Abstract

**Objective:**

The main aim of this study was to assess the magnitude of malnutrition and associated factors among HIV infected children in Amhara Regional State, and Northwest Ethiopia. This study is a result of single observation.

**Results:**

A total of 372 study participant were interviewed, of which 60.2%, 95% CI (55.90–65.60) were malnourished. Children who had good individual dietary diversity were 53% times less likely malnourished as compared to children who had fair/poor dietary diversity (AOR = 0.474, 95% CI (0.26, 0.86)). Sex of child, Age of the child, undiversified diet, comorbidity disease, oral ulcer, diarrhea and history of hospital admission were found statically significant associated with malnutrition. Magnitudes of malnutrition among HIV infected children were very high. The early detection and control progression of HIV, closely follow up to intervene this situation is highly recommended.

## Introduction

Worldwide malnutrition is the major underlying cause of death in children [1]. Each year it claims the lives of over one million children were dead and develop severe acute malnutrition. Among children with severe malnutrition, mortality risk is three times higher in HIV-infected children than in non-HIV-infected children [2, 3].

The harmful effect of malnutrition is more common in HIV infected children, For example, wasting and weight loss are common features of HIV infected children [4, 5]. Use of ART without nutritional support, or nutritional support without ARTs, will often result in poor treatment responses and outcomes [6].

So WHO recommended that asymptomatic HIV infected children should increase his/her energy intake by 10%, compared to a non-infected child but if they were symptomatic HIV infection or episodes of opportunistic infection developed also added to up to 20–30% and when a severe malnutrition episode occurs added up to 50–100% [7]. The study which is conducted in Bangladesh the magnitude of malnutrition in HIV infected children differs from males to females. These showed that 33.2% males and 26.7% of females were malnourish and it was similar in Addis Ababa, Ethiopia [8, 9].

However, despite the seriousness of the problem, the number of studies conducted to explain factors that contribute to malnutrition among HIV infected children is still relatively inadequate particularly programmatic implications are not paid attention. This is the main reason for undertaking this research hoping that it will shed some light on the issue and provide important information both to the clinicians and to the programmers to manage malnutrition among HIV infected children.

## Main text

### Methods

The study used institutional based comparative cross-sectional study was conducted in three public hospitals. It was conducted at Finote Selam primary hospital, Shegaw Motta primary hospital and Debre Markos Referral Hospital in Amhara Regional State from February 20, 2017 to April 20, 2017. These three selected sites were serving more than 1 million, 990,000 and 3.5 million people respectively. In Debre Markos referral hospital, 326 were HIV infected children of which 168 male and 158 female. In Finote Selam primary hospital, 127 were HIV infected children of which 63 males and 64 females and from 990,000 people 128 were HIV infected of which 58 males and 70 females HIV/AIDS infected children had followed up care respectively [10–12]. The sampled patients (age 2–14 years) who tested positive for HIV/AIDS and were being routinely followed for HIV care or sooner as the clinical providers. The sample size was determined using a difference between two population proportions, considering the following conditions [13]:

1. The proportion of HIV infected wasted children of male 12.9% and of female 34.9%.
2. The proportion of HIV infected underweight children of male 24.7% and of female 16.4%.
3. The proportion of HIV infected stunted children of male 34.3% and of female 26.2%.
4. With 5% level of significance (α), at 95% level of confidence for two tail test, r is the ratio of the size of sample 2 to sample 1 = 1 and a Power of (1 − B)) = 80%. The required sample size is calculated as follows:

$$let\;\;\;r = \frac{{n_{2} }}{{n_{1} }}\;{\text{and}}\;{\text{P}} = \frac{{{\text{n}}_{ 1} p_{1} + {\text{n}}_{ 2} p_{2} }}{{{\text{n}}_{ 1} + n_{2} }} \, = \frac{{p_{1} + {\text{r}}p_{2} }}{ 1+ r}\;{\text{is}}\;{\text{the}}\;{\text{common}}\;{\text{population}}\;{\text{proportion}}$$

$${\text{n}}_{ 1} = \frac{{\left[ {Z_{{{\raise0.7ex\hbox{$\alpha $} \!\mathord{\left/ {\vphantom {\alpha 2}}\right.\kern-0pt} \!\lower0.7ex\hbox{$2$}}}} \sqrt {\left( {1 + \frac{1}{r}} \right) \, p \, (1 - p)} + Z_{\beta } \sqrt {p_{1} (1 - p_{1} ) + \left( {\frac{{p_{2} (1 - p_{2} )}}{r}} \right)} } \right]^{2} }}{{\left( {p_{1} - p_{2} } \right)^{2} }} \,$$

Taking the largest sample size 1028 obtained for wasting from the above calculation and correcting for a finite population of size 581 target populations, the required sample size is 372 (186 male and 186 female).

The sampling procedure was a total of 581 HIV/AIDS positives children who had to follow up of the ART care in the study facilities, 372 participants were proportionately selected each study site. A systematic sampling technique was used to select the participants for an interview, anthropometric measurement and clinical records review from the register of each hospital. Sampling interval (Kth) was determined by dividing the total HIV/AIDS positive males and females’ children in each hospital allocated sample size of each hospital males and females’ children. For DMRH k1 = 168/108 = 2 and K2 = 158/100 = 2, SMPH k1 = 58/38 = 2 and K2 = 70/45 = 2, FSPH k1 = 63/40 = 2 and 64/41 = 2 where k1 = male and K2 = female. The first sample was selected by simple random sampling from clinical records and every (kth) record was selected for gathering information until the required sample was obtained from each Hospital.

Data collectors were recruited and conducted 2-day training by principal researcher. Anthropometric measurements, record review, and face to face interview were conducted. Weight and height/were measured for each subject. During data collection, nutritional advice was provided to all caregivers and who had a malnourished child were linked to the therapeutic feeding program. The pre-test was conducted at Debre Markos health center. During data collection, all the activities were carefully monitored and supervised. Weights and height/were measured using standardized tools and calibration was applied daily. The questionnaire was prepared in English and translated to Amharic.

Operational definitions used in this study are malnutrition: is defined WAZ or WHZ or HAZ or BAZ below − 2 SD of the median or WAZ or WHZ or HAZ or BAZ above 2 SD value of WHO standard respectively.

Data processing and analysis; data were entered into EPI DATA version 3.1 and analysis were done using SPSS version 20 statistical package and WHO child growth standards 2007 software. The anthropometric measurement was converted physical growth indicators (WHZ, WAZ, HAZ and BAZ) and was used WHO anthro and WHO anthroplus software. Logistic regressions analysis was computed to assess the associations of the various factors against the level of malnutrition. Variables with a p-value of less than 0.2 in the bivariate analysis were entered into the final model. A p-value < 0.05 at 95% CI was considered statistically significant. The strength of association between associated factors and outcome variables were assessed using odds ratio. Since all study participants were children, their parents or guardians gave informed consent.

### Results

Socio-economic characteristics given below as a total of 372 HIV/AIDS infected children were a participant in the study with the response rate of 100%. The magnitude of malnutrition among HIV/AIDS infected Children were; totally 224 (60.2%) HIV/AIDS infected children were malnourished among the studied children.

Among the children studied, 186 (50%) and 186 (50%) were males and females respectively. among the children studied, 126 (67.7%) and 98 (52.7%) were malnourished males and females respectively, 52 (96.3%) were found in age group of 2–5 year, 72 (48.3%) were found in age group of 5–10 year and 100 (59.2%) were found in age group of 10–15 years. The mean (± SD) age of children included in the study was 9.6 (± 3.2) years ranging from 2 to 14 years (Table 1).

**Table 1:** socioeconomic characteristics of HIV infected children who had to follow up in three public hospitals, Northwest, Ethiopia, 2017

Variables	Categories	Frequency	Percent
Sex	Male	186	50
	Female	186	50
Age of child (years)	2–5	54	14.5
	5–10	149	40.1
	10–15	169	45.4
A child with alive mother	Yes	297	79.8
	No	75	20.2
Age of mother	15–24	8	2.2
	25–34	122	32.8
	35–49	159	42.5
	≥ 50	9	2.4
Residence	Rural	82	22
	Urban	290	78
Occupation of mother (n = 297)	Student	2	0.5
	Daily laborer	102	27.4
	Employed	53	14.2
	Farmer	51	13.7
	Merchant	62	16.7
	Other	27	7.7
School attended (N = 297)	Yes	142	47.8
	No	155	52.2
Level of maternal education	Unable to read and write	99	26.6
	Able to read and write	53	14.2
	Primary school	58	15.6
	Secondary school	44	11.8
	College/university	43	11.6

Dietary characteristics of study participant, 176 (47.7%) reported good food variety and 196 (52.3%) ate also fair/poor food variety per 24 h.

Clinical characteristics of HIV infected children. A total of 372 (100%) the studied children, 343 (92.2%) were received ART for greater than 12 months. Among the children studied 195 (52.4%) and 106 (68%) of which had WHO stage of (I and II) during the survey time respectively and 253 (28.5%) and 87 (23.4%) also had WHO stage of (I and II) the past 6 months respectively, whereas 15.4% and 20.8% of were had CD4 count less than 500 cell/mm^3^ and 84.6% and 79.2% were above 500 cell/mm^3^ during the surveys time respectively. More than 1/3rd (177) of the study participant was developed an oral ulcer in the last 6 months and among them 109 (61.6%) were malnourished.

Associated factors of malnutrition among HIV infected children; Children who were male 2.3 times more likely developed malnutrition as compared to female AOR = 2.37 (1.34, 4.20). Age of child who was between 5 and 10 years were 96% times less likely developed malnutrition as compared to age between 2 and 5 years. Whereas age of a child between 10 and 15 year was 95.5% time less likely developed malnutrition as compared to age of between 2 and 5 years. Children who had good individual dietary diversity were 53% times less likely develop malnutrition as compared to children who had fair/poor dietary diversity (AOR = 0.47, 95% CI (0.26, 0.86)). In addition, children who had diarrhea that stays for 2 weeks in the last 6 months were 3.3 times more likely to be malnourished as compared who had no diarrhea (AOR = 3.30 CI (1.41, 7.72)). Children who had an ulcer oral also were 2.3 times more likely develop malnutrition as compared to children who had no oral ulcer (AOR = 2.30, 95% CI (1.41, 7.60)). Children who had no comorbidity disease were 65.2 times less likely developed malnutrition, as compared to these, had comorbidity disease (AOR = 0.34, 95% CI (0.15, 0.800)). Children who had no history of hospital admission were also 66.4% times less likely malnutrition as compared to who had a history of hospital admission (AOR = 0.34, 95% CI (0.13, 0.84)) (Table 2).

**Table 2:** Multivariate analysis of selected variables associated factor for malnutrition in HIV/AIDS infected children on HIV-care who had followed up in three public Hospitals, northwest, Ethiopia

Variables	Categories	Malnutrition		COR 95% CI	AOR 95% CI
		Yes	No		
Sex of the child	Male	126 (60.2)	60 (32.3)	1.89 (1.24, 2.87)	2.37 (1.34, 4.20)
	Female	98 (52.7%)	88 (47.3)	1	1
Age of child (years)	2–5	52 (96.3%)	2 (3.7%)	1	1
	5–10	72 (48.3%)	77 (51.7%)	0.04 (0.01, 0.15)	0.04 (0.01, 0.21)
	10–15	100 (59.2%)	69 (40.8%)	0.06 (0.01, 024)	0.05 (0.01, 0.25)
Dietary diversity	Good	110 (62.5%)	66 (37.5%)	1	1
	Fair/poor	114 (58.2%)	82 (41.8%)	1.20 (0.79, 1.82)	0.47 (0.26, 0.86)
Presences of co morbidity disease	Yes	39 (53.4%)	34 (46.6%)	0.70 (0.42, 1.18)	0.34 (0.15, 0.80)
	No	185 (61.9%)	114 (38.1%)	1	1
Diarrhea	Yes	58 (65.9%)	30 (34.1%)	1.37 (0.80, 2.27)	3.30 (1.41, 7.72)
	No	166 (58.5%)	118 (45.5%)	1	1
Ulcer of oral	Yes	109 (61.6%)	68 (58.4%)	1.12 (0.74, 1.69)	2.30 (1.41, 7.60)
	No	115 (59%)	80 (41%)	1	1
Hospital admission	Yes	40 (58%)	29 (42%)	1.12 (0.66, 1.91)	0.34 (0.13, 0.84)
	No	184 (60.7%)	119 (39.3%)	1	1

### Discussion

This study investigated the magnitude and associated factors of all types of malnutrition and associated factors among HIV infected children in Northwest Ethiopia.

About three out of five HIV positive children were malnourished. The finding implied that HIV infected children in the current study area were in the worst condition compared to malnutrition reported in Nigeria [14]. This could probably attribute the difference in the characteristics of study participants. On the other hand, the current prevalence was similar in a study Bobo Dioulasso City, Burkina Faso and Cameroon [23, 24]. Thus, malnutrition is still a major public health problem in Africa in general and in Ethiopia in particular.

Boys were more liekly to be malnourished compared to girls. Such finding was similar with study in Tanzania among children born to HIV-infected mothers; boys were more likely affected. About 28% were stunted, 40% were wasted and 28% were underweight compared to girls [8]. This can be explained that boys need more close investigation of malnutrition and appropriate management is needed. The reason might be due to sex preference and unbalanced care of children based on their sex [15]. Yet, the reasons for this association between gender and malnutrition have to be explored using qualitative study in order to explore feeding practice based on gender difference [9]. Those children older than 5 years and beyond were less likely to be malnourished compared those below 2 years. The reason can be the older children adapted feeding situation and opportunistic infections are managed. However, it was contracting with other studies that found older children were more likely to experience malnutrition [16, 17]. The discrepancy can be explained as young children metabolic needs for growth and development is higher than older once. This hypothesizes that the younger children are prone to fast in the HIV-disease progression but with higher needs of caloric intake [1, 18]. Hence, children less than 2 years need special attention in order to ensure adequate energy requirement was met.

In the current study children who had diarrhea were 3.3 times more likely developed malnutrition than who had not diarrhea. This is similar to other studies elsewhere [19, 20]. This can be explained due to alteration of gastro intestine tract function or malabsorpation of food. Similarly, those who had oral ulcer had also statically significant associated with malnutrition. These findings were in line with the studies conducted in other areas revealed those with gastro intestine (GI) disorders were associated with malnutrition in HIV infected patients [4, 21–25]. This could be due to difficulty of eating or chewing. In addition, the disease could be brought loss of appetite and decrease peristalsis of gastro intestine tract. This implied that opportunistic infections have to be managed and symptomatic treatment is necessary to restore food intake of children with GI symptoms.

The strength of this study is it balanced male and female children, it is conducted in low resource setting and it give information on the linkage of HIV and malnutrition among children. Further prospective study is recommended to observe nutritional status overtime. Qualitative study may also help to investigate the reason why male were more malnourished than females? Finally, policy makers can use the information to improve HIV and nutrition interventions based on the result.

## Limitations of the study

Since the study used cross sectional study,

- It cannot establish the cause effect relationship between the dependent and the independent variable. Hence, prospective study design supplemented with qualitative approach will solve the limitations of this study.

## Abbreviations

AIDS: acquired immune deficiency syndrome
ART: anti retro viral therapy
CD4: cluster differentiation of T-lymphocyte cells
HIV: human immune deficiency virus
IDD: individual dietary diversity
WHO: World Health Organization
